# Partnering With the Community to Develop and Pilot Test a Culturally Tailored Dementia Education Program

**DOI:** 10.1093/geroni/igaf122.550

**Published:** 2025-12-31

**Authors:** Manka Nkimbeng, Truphosa (Posa) Aswani, Wynfred Russell, Tetyana Shippee, Joseph Gaugler

**Affiliations:** University of Minnesota School of Public Health, Minneapolis, Minnesota, United States; University of Washington, Seattle, Minnesota, United States; Strengthening African Resilience Towards Excellence, Brooklyn Park, Minnesota, United States; University of Minnesota, Minneapolis, Minnesota, United States; University of Minnesota, Minneapolis, Minnesota, United States

## Abstract

The Immigrant Memory Collaborative is a community university partnership that leveraged community needs and asset assessment to develop a culturally tailored dementia education program with the African immigrant community in Minnesota. Guided by a project advisory board, key informant interviews and community conversations informed the content of African MaDE: Memory and Dementia Education, by and for the African community. This presentation will describe the community engagement approach used to pilot test African MaDE with the community and pre-post evaluation. In addition to the primary partner, the team leveraged its network to collaborate with community organizations to host education sessions for 187 unique attendees at 11 events in person and remotely. Of these, 77 participants completed pre- and post-dementia knowledge questions. Most (55) attendees were first-generation (born outside the United States). Most of these participants were neither current nor past care partners and had never provided care for a person living with dementia in a professional capacity. Preliminary findings show a statistically significant 0.9-point improvement in dementia knowledge from pre- to post-education. Qualitative comments also reveal that participants appreciate the presentation and content. African MaDE is feasible and acceptable to the African community. We are currently using a community-engaged approach to conduct large-scale dissemination of this program. In addition to describing our process, we will present lessons learned in this community-engaged education development that can inform the development of similar programs in other communities.

